# Nutrition Support and Tight Glucose Control in Critically Ill Children: Food for Thought!

**DOI:** 10.3389/fped.2018.00340

**Published:** 2018-11-06

**Authors:** Vijay Srinivasan

**Affiliations:** ^1^Department of Anesthesiology and Critical Care Medicine, The Children's Hospital of Philadelphia, Philadelphia, PA, United States; ^2^Department of Anesthesiology and Critical Care, Perelman School of Medicine at the University of Pennsylvania, Philadelphia, PA, United States

**Keywords:** tight glucose control, intensive insulin therapy, enteral nutrition, parenteral nutrition, children, critical illness, outcomes

## Abstract

Numerous studies have examined the strategy of tight glucose control (TGC) with intensive insulin therapy (IIT) to improve clinical outcomes in critically ill adults and children. Although early studies of TGC with IIT demonstrated improved outcomes at the cost of elevated hypoglycemia rates, subsequent studies in both adults and children have not demonstrated any benefit from such a strategy. Differences in patient populations, variable glycemic targets, and glucose control protocols, inconsistency in attaining these targets, heterogeneous intermittent sampling, and measurement techniques, and variable expertise in protocol implementation are possible reasons for the contrasting results from these studies. Notably, differences in modes of nutrition support may have also contributed to these disparate results. In particular, combined use of early parenteral nutrition (PN) and a strategy of TGC with IIT may be associated with improved outcomes, while combined use of enteral nutrition (EN) and a strategy of TGC with IIT may be associated with equivocal or worse outcomes. This article critically examines published clinical trials that have employed a strategy of TGC with IIT in critically ill children to highlight the role of EN vs. PN in influencing clinical outcomes including efficacy of TGC, and adverse effects such as occurrence of hypoglycemia and hospital acquired infections. The perspective afforded by this article should help practitioners consider the potential importance of mode of nutrition support in impacting key clinical outcomes if they should choose to employ a strategy of TGC with IIT in critically ill children with hyperglycemia.

## Introduction

Clinical trials of tight glucose control (TGC) with intensive insulin therapy (IIT) to improve outcomes in critically ill adults and children have promised much, but delivered little. While the first studies in critically ill adults in surgical intensive care units (ICUs) demonstrated improvements in mortality and morbidity from TGC with IIT (1, 2), later studies in medical and mixed medical/surgical ICUs observed worse outcomes (3–6). Disappointingly, TGC with IIT resulted in substantial increases in hypoglycemia in all these studies with corresponding poor clinical and neurological outcomes (7–9). Similarly, in critically ill children, the first study of TGC with IIT was notable for significant decreases in length of stay and inflammation, but came at a cost of substantial increase in hypoglycemia rates (10). Subsequent studies in critically ill children were unable to demonstrate any benefits from TGC with IIT, and continued to report elevated hypoglycemia rates in spite of measures such as continuous glucose monitoring (CGM) and computer guided decision making to reduce hypoglycemia (11–13). Follow-up neurodevelopmental studies have observed worse clinical outcomes from hypoglycemia due to TGC with IIT in critically ill children (14, 15). Differences in patient populations, variable glycemic targets and glucose control protocols, inconsistency in attaining these targets, heterogeneous intermittent sampling and measurement techniques, and variable expertise in protocol implementation are possible reasons for the contrasting results from these studies (16–18). Notably, differences in modes of nutrition support may have also contributed to these disparate results. In particular, combined use of early parenteral nutrition (PN) and TGC with IIT may be associated with improved outcomes, while combined use of enteral nutrition (EN) and TGC with IIT may be associated with equivocal or worse outcomes. This article will examine how mode of nutrition support may influence blood glucose (BG) concentrations, and provide perspectives on how nutrition support may influence the efficacy of TGC with IIT and occurrence of adverse events.

## Stress hyperglycemia in pediatric critical illness

Stress hyperglycemia commonly occurs in critically ill children, even in those with previously normal glucose homeostasis (19–25). Over two-thirds of critically ill children experience moderate hyperglycemia [BG concentrations > 150 mg/dL (> 8.3 mmol/L)], while severe hyperglycemia [BG concentrations > 200 mg/dL (> 11 mmol/L)] occurs in as many as one-third of critically ill children (19–25). Stress hyperglycemia develops via a combination of increase in gluconeogenesis (relative to glucose uptake and turnover) and development of insulin resistance (26). Both of these mechanisms are mediated by increases in inflammatory cytokines as well as elevated levels of counter-regulatory hormones (catecholamines, cortisol, glucagon and growth hormone) (27, 28). Additional mechanisms for stress hyperglycemia include impairments in pancreatic beta-cell function with corresponding reduction in insulin secretion (29).

The mode of nutrition support in the ICU can also exacerbate stress hyperglycemia (30). Critically ill children are often prescribed PN due to inability to tolerate EN in critical illness states. The provision of excess carbohydrate calories in PN can result in elevated BG concentrations. While normal infants and children may have substantially higher glucose turnover rates than adults (31), limited data from critically ill children suggest that glucose infusion rates (GIR) < 5 mg/kg/min may be optimal for glucose utilization from PN (32, 33). The practice of cycling PN may also be associated with stress hyperglycemia, most likely due to impaired insulin secretion (34). Commonly used predictive equations to calculate energy expenditure needs in critical illness states are inferior to targeted indirect calorimetry, and often result in over prescription of calories (35–37). In contrast, nutrition strategies such as supplementation of PN with glutamine and the administration of low calorie PN may reduce the development of stress hyperglycemia during critical illness (38, 39).

In turn, stress hyperglycemia can affect the delivery of nutrition during critical illness in a variety of ways. Stress hyperglycemia may influence the ability to provide consistent or adequate EN during critical illness due to delayed gastric emptying and slowing down of gut motility (40). Stress hyperglycemia can also impair the prokinetic action of erythromycin on gastric emptying (41). Altered gut motility and insensitivity to prokinetic agents may result in intolerance to EN. Studies in critically ill adults have demonstrated the association of intolerance to EN with stress hyperglycemia and BG variability (42). Stress hyperglycemia also results in altered nutrient utilization during critical illness. Stress hyperglycemia exacerbates protein catabolism in skeletal muscle in critically ill adults with severe burns (43). Stress hyperglycemia may also reduce the activity of lipoprotein lipase contributing to the development of hypertriglyceridemia through reduced clearance of circulating triglycerides (44).

Stress hyperglycemia during critical illness thus results in the rapid availability of glucose as a fuel for metabolic processes occurring in vital organs in the body. During the acute phase of critical illness coinciding with high metabolic demands, stress hyperglycemia may be adaptive to favor survival. However, during the chronic phases of critical illness, persistence of stress hyperglycemia may reflect impaired allostasis with potential for harm from increased oxidative damage due to propagation of the proinflammatory response, and impaired cellular repair and tissue healing (30). Though stress hyperglycemia is often justified as an adaptive response to critical illness (45, 46), numerous studies in critically ill children have observed the association of stress hyperglycemia with poor clinical outcomes across a variety of disease states (19–25, 47–53). Consequently, the strategy of TGC with IIT emerged as a viable and rational solution to improve outcomes in critically ill children experiencing stress hyperglycemia.

## TGC with IIT in critically ill children

Early studies of TGC with IIT in critically ill children focused on children with severe burns and very low birth weight neonates (54–56). Subsequently, this practice of TGC with IIT was studied in more general populations of critically ill children with varying results (10–13). The variability in observed outcomes across these studies are due to several important methodological and epidemiological differences that are summarized in Table 1. In the study by Vlasselaers et al, BG concentrations were controlled to age-specific fasting ranges in the intervention group that were substantially lower than the BG ranges in the intervention groups in the other three studies (Safe Pediatric Euglycemia after Cardiac Surgery—SPECS, Control of Hyperglycaemia in Pediatric Intensive Care—CHiP and Heart and Lung Failure—Pediatric Insulin Titration—HALF-PINT). The control group BG range also differed between these studies. Additionally, there was greater separation of BG concentrations in the study by Vlasselaers et al, compared to the SPECS, CHiP, and HALF-PINT studies. Another important difference between these studies was the striking variation in overall incidence of acquired infections in study subjects. The study by Vlasselaers et al. observed a much higher incidence of acquired infection in the control group than SPECS, CHIP, or HALF-PINT which could possibly reflect important definitional and epidemiological differences compared to the latter three studies. Consequently, the trial by Vlasselaers et al. was more likely than the other three trials to have identified a positive benefit from TGC with IIT.

**Table 1:** Studies of tight glycemic control with intensive insulin therapy in critically ill children—demographics and methodology.

**Study**	**Sample size (*n*)**	**Number of centers**	**Age range (years)**	**Diagnosis categories**	**TGC range vs. control range (BG, mg/dL)**	**Blood glucose management details**	**Primary outcome in TGC range vs. control range**
Vlasselaers et al. (10)	700	1	0–16	75% cardiac surgery; 25% medical-surgical	<1 year old: 50-80 vs. >215; >1 year old: 70–100 vs. >215	Paper based guideline; arterial blood samples; blood gas analyzer	Clinical: Days in ICU (5.51 vs. 6.15, *p* = 0.017) Biochemical: CRP change from baseline to Day 5, mg/L (−6 vs. 0, *p* = 0.007)
Agus et al. (11)	980	2	0–3	Cardiac surgery	80–110 vs. standard care	CHECKS algorithm; arterial blood samples; CGMS/POC	30 days rate of healthcare associated infections—number of infections per 1,000 patient-days in the cardiac ICU (8.6 vs. 9.9, *p* = 0.67)
Macrae et al. (12)^*^	1369	13	0–16	61% cardiac surgery; 39% medical-surgical	72–126 vs. 180-215	Paper based guideline; arterial blood samples; POC	Number of days alive and free from mechanical ventilation at 30 days (23.6 vs. 23.2, mean difference = 0.36, 95% CI−0.42–1.14)
Agus et al. (13)^*^	713	35	0–17	Medical-surgical	80–110 vs. 150–180	CHECKS algorithm; arterial blood samples; CGMS/POC	Number of ICU free days through Day 28 (20 vs. 19.4, *p* = 0.86)

* *Study stopped early*.

*TGC: tight glycemic control; BG: blood glucose; ICU: intensive care unit; CRP, C-reactive protein; CI, confidence intervals; CHECKS, Children's Hospital Euglycemia for Kids Spreadsheet; CGMS: continuous glucose monitoring sensor; POC, point of care*.

Published meta-analyses of studies of TGC with IIT in critically ill children have not observed any benefits from this strategy (57–59). In 2014, Srinivasan et al. carried out the first systematic review and quantitative meta-analysis of the 4 studies till date that had examined the efficacy and safety of TGC with IIT in critically ill children (excluding neonates) (57). This meta-analysis observed that TGC with IIT did not reduce 30-day mortality, but did appear to reduce acquired infection in critically ill children at the expense of higher incidence of hypoglycemia. Subsequently, Zhao et al. published an updated meta-analysis in 2018 including the results from the recent HALF-PINT trial and observed that TGC with IIT did not reduce 30-day mortality or acquired infection in critically ill children, but resulted in substantial increases in hypoglycemia rates (58). Recently, Chen et al. carried out a meta-analysis of 6 studies of TGC with IIT in critically ill children and preterm neonates, and concluded that the practice of TGC with IIT did not confer any benefits but did result in significant increases in hypoglycemia (59). Notably, none of these above meta-analyses took into account mode of nutrition support when evaluating the safety and efficacy of TGC with IIT in critically ill patients.

## Modes of nutrition support: implications for efficacy and safety of TGC with IIT

### Data from adult studies

In the single-center surgical and medical adult ICU studies of TGC with IIT from Leuven, early use of PN for nutrition support was heavily favored during ICU admission (1, 3). Per existing European Society of Parenteral and Enteral Nutrition (ESPEN) guidelines at the time of these studies, the use of early PN to reach goal energy needs within 3 days of admission was emphasized if they were not expected to tolerate EN or had a contraindication to use of EN (60). In the original Leuven surgical ICU study from Leuven, subjects received on an average 1,100 kcal/day. The majority of energy delivery was via PN started on the first day of ICU admission (768 kcal/day in the form of 20% intravenous dextrose solution from the first day onwards) (1). Similarly, in the subsequent Leuven medical ICU study, the majority of energy delivery was from PN started on the first day of ICU admission (ranging between 750 and 800 kcal/day) (3). In contrast, the NICE-SUGAR study, in accordance with customary practice in Australia, New Zealand and Canada at the time of the study, almost exclusively relied on the use of EN for nutrition support during ICU admission to provide on average 880 kcal/day with goal energy needs reached by 7–10 days (7). In the NICE-SUGAR study, PN was used to supplement EN delivery and energy from PN never exceeded 300 kcal/day.

### Data from pediatric studies

In the pediatric Leuven study (similar to the adult Leuven studies), PN was started at ICU admission (in the form of 20% intravenous dextrose solutions) while attempts were made to start EN as soon as feasible based on the underlying condition (10). On average, infants received 42 kcal/kg/day and children received 28 kcal/kg/day during their ICU admission with substantial energy delivery via exclusive PN in over 40% of the study population (and PN with partial EN in the remaining 60%). To a lesser extent than the pediatric Leuven study, the SPECS trial also relied predominantly on PN for energy delivery in over 50% of the study population (11). During the period of study enrollment in the SPECS trial, subjects in the TGC group received a median of 41% of their total caloric intake via EN, while 38% of subjects in the control group received a median of 38% of their total caloric intake via EN. In contrast, the CHiP and HALF-PINT trials largely relied on EN for energy delivery based on current clinical practice guidelines at the time of the studies (12, 13). In the CHiP trial, by Day 7 of enrollment, the median enteral caloric intake was approximately 20 kcal/kg/day and the median parenteral caloric intake was approximately 5 kcal/kg/day in enrolled subjects (12). In the HALF-PINT trial, by Day 7 of enrollment, median EN delivery was approximately 25 kcal/kg/day which accounted for approximately 60% of total energy delivered in enrolled subjects (13). Table 2 summarizes key differences in nutrition support, measures of glycemia, and primary outcomes with effect size across these pediatric studies.

**Table 2:** Studies of tight glycemic control with intensive insulin therapy in critically ill children—nutrition support and glycemia.

**Study**	**Nutrition protocol for study**	**Details of nutrition support**	**Median daily caloric intake (kcal/kg/day)**	**Average daily insulin dose in TGC range vs. control range (IU/kg/day)**	**Time weighted average BG in TGC range vs. control range (mg/dL)**	**Hypoglycemia in TGC range vs. control range (BG < 40 mg/dL), %**
Vlasselaers et al. (10)	Yes	Early PN (20% dextrose with 10% amino acids); exclusive PN in >40% of subjects and PN with partial EN in ~60% of subjects	Infants: 42 Children: 28	1.3 vs. 0.0	113 vs. 158	25 vs. 1
Agus et al. (11)	No; local site practice	PN in >50% of subjects; partial EN in 56% in TGC group and 59% in control group	50	0.2 vs. 0.0	112 vs. 121	3 vs. 1
Macrae et al. (12)	No, local site practice	EN and PN per local site practice	25	0.18 vs. 0.07	107 vs. 114	7.3 vs. 1.5
Agus et al. (13)	Yes	Early EN; EN in 53% of TGC group and 55% in control group	40	0.74 vs. 0.01	109 vs. 123	5.2 vs. 2

*TGC, tight glycemic control; BG, blood glucose; PN, parenteral nutrition; EN, enteral nutrition*.

### Interaction of TGC with IIT and mode of nutrition support (EN vs. PN)

The mode of nutrition support (EN or PN) is a key variable that has important implications for the efficacy and safety of TGC with IIT in both adults and children. In the single center Leuven adult studies, the aggressive early initiation of PN to rapidly reach goal energy needs by 48–72 h following admission may have aggravated the problem of stress hyperglycemia and risk of worse outcomes in a population of critically ill adults with high prevalence of pre-existing diabetes mellitus. Consequently, the strategy of TGC with IIT may have proven beneficial in this setting (61). In contrast, use of TGC with IIT in the relatively nutrition restricted setting of NICE-SUGAR may have been harmful by evoking a global substrate deficit via insulin-induced suppression of proteolysis, lipolysis, glycogenolysis, and gluconeogenesis. By suppressing these important compensatory mechanisms which play a vital role in states of energy deprivation and starvation, TGC with IIT likely resulted in worse outcomes in this large study that relied predominantly on EN (61).

A published meta-analysis by Marik et al. in 2010 took into account mode of nutrition support (EN vs. PN) using meta-regression techniques to control for proportion of intravenously delivered calories from PN (62). This meta-analysis demonstrated reduced mortality from TGC with IIT when PN was utilized as the predominant mode of nutrition support. After excluding the two Leuven trials that predominantly employed PN as the mode of nutrition support, the authors observed that mortality was lower with control patients receiving usual care with EN suggesting that TGC with IIT may be harmful in patients receiving exclusive or predominant EN (62).

**Figure 1:**
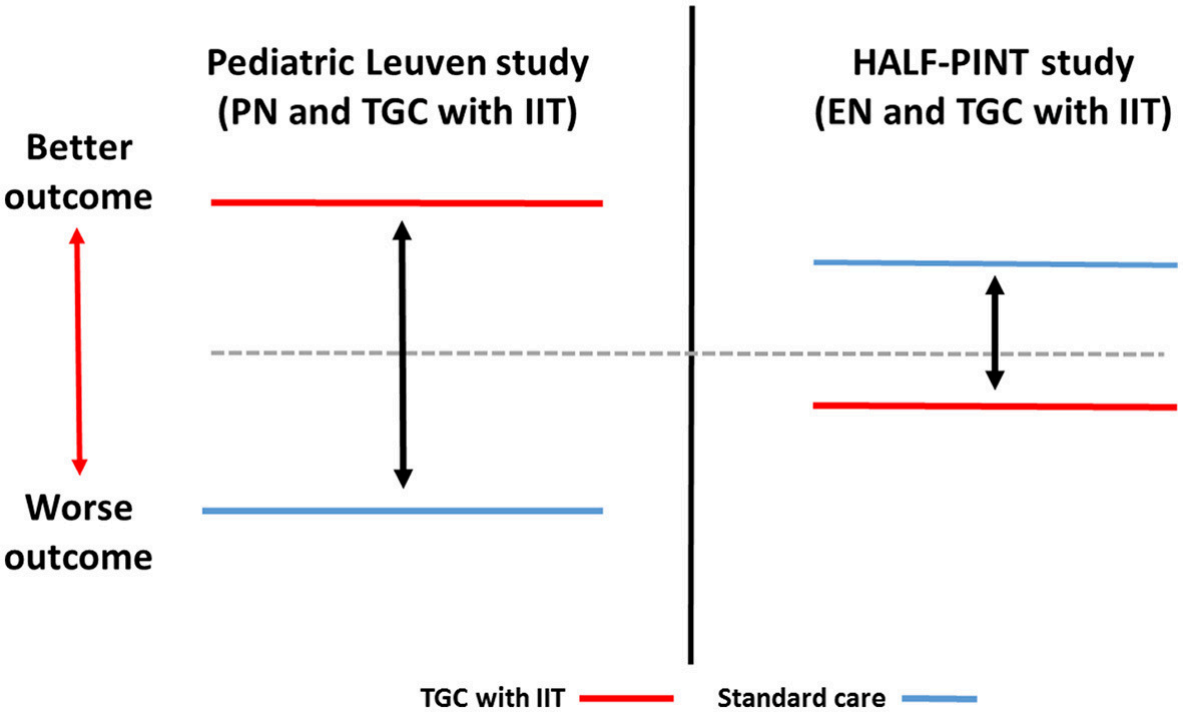
Schematic comparison of interaction of TGC with IIT and mode of nutrition support (EN vs. PN) with impact on outcomes in critically ill children. Pediatric Leuven study: Vlasselaers et al. (10); HALF-PINT study: Agus et al. (13). TGC with IIT, Tight glucose control with intensive insulin therapy; EN, enteral nutrition; PN, parenteral nutrition.

In published studies of TGC with IIT in critically ill children till date, less granular information is available regarding timing, intensity, duration and mode of nutrition support. This limits our ability to provide detailed analyses of interaction of mode of nutrition support and TGC with IIT on clinical outcomes similar to adult studies. Similar to the adult studies, the pediatric studies also vary in nutrition support practice which makes direct comparisons challenging. The pediatric Leuven study demonstrated benefits of TGC with IIT coupled with a nutrition support strategy that relied mainly on early PN (10). In contrast, the HALF-PINT study predominantly emphasized EN delivery and observed trends to better outcomes with usual care targeting BG of 150–180 mg/dL (8.3–10 mmol/L) (13). The schematic comparison of differences in mode of nutrition support (EN vs. PN) and potential impact on TGC with IIT to influence outcomes is depicted in Figure 1 using the example of the pediatric Leuven and HALF-PINT studies.

The important question for practitioners is whether any one mode of nutrition support—EN or PN—is superior to another to improve outcomes in critically ill adults and children. In critically ill adults, early EN initiation compared to early PN initiation did not reduce mortality or reduce secondary infections, but was associated with more digestive complications and hypoglycemia (63, 64). Based on current evidence, use of PN is feasible and appears to result in rapid increase in energy and protein delivery with the ability to reach target goals early on during critical illness (65). In contrast, use of EN is often delayed due to illness severity and frequently does not reach goal energy and protein needs due to recurrent interruptions to enteral feeding (66). Early initiation of EN (compared to delayed initiation of EN) appears to be associated with improved clinical outcomes in critically ill adults and children (67, 68). However, it was not clear until recently if early initiation of PN compared to delayed initiation of PN was beneficial or harmful in critically ill subjects. This vital question of timing of PN initiation (early vs. delayed) was addressed in critically ill adults and children by the Early Parenteral Nutrition Completing Enteral Nutrition in Adult Critically Ill Patients (EPaNIC) study and the Early vs. Late Parenteral Nutrition in the Pediatric Intensive Care Unit (PEPaNIC) study, respectively (69, 70). In both the adult and pediatric studies, delayed PN initiation compared to early PN initiation was associated with better clinical outcomes in the form of fewer acquired infections and lower ICU dependency. There were no mortality differences between the two groups in either study. In both studies, TGC with IIT was employed to maintain normoglycemia. Notably, the delayed PN initiation group experienced more hypoglycemia episodes in both studies, likely due to greater proportion of nutrition support in the form of EN provided to this group during study enrollment coupled with a strategy of TGC with IIT. This finding of more frequent hypoglycemia episodes in the delayed PN initiation group is similar to other studies of TGC with IIT in critically ill patients that have relied predominantly on EN for nutrition support (6, 12, 13). Interestingly, even though the delayed PN initiation group experienced fewer infections, there was more inflammation (as measured by C-reactive protein levels) in this group compared to the early PN initiation group in both studies (69, 70). This raises the intriguing possibility that a strategy that relies predominantly on EN might favor an uncoupling of the inflammatory state from processes involved in autophagy and maintenance of the gut function to improve clinical outcomes (71–73).

## Conclusion

In summary, studies of TGC with IIT have demonstrated varying results due to numerous methodological and target population differences, but an important factor that may often go underappreciated is the mode of nutrition support in the form of either EN or PN, or a combination of the two. Specifically, when PN is the favored mode of nutrition support, TGC with IIT may be associated with improved outcomes compared to usual care, as usual care in this setting of early PN may be associated with more harm from uncontrolled hyperglycemia. In contrast, when EN (or predominantly EN) is the favored mode of nutrition support, TGC with IIT may be associated with equivocal or worse outcomes compared to usual care, as usual care in this setting of early EN may be more beneficial with maintenance of gut function and processes involved in autophagy. While pediatric data surrounding the interaction of mode of nutrition support and TGC with IIT is limited, the practitioner should consider the potential importance of mode of nutrition support in impacting key clinical outcomes, if they should choose to employ a strategy of TGC with IIT to manage critically ill children. Future studies of TGC with IIT should use targeted indirect calorimetry for more accurate estimation of energy needs, and ensure targeted protein delivery to meet minimum threshold goals. Such studies should also incorporate better decision making support for nutrition delivery and glucose control in the form of protocol-driven algorithms and continuous glucose monitoring technologies to ensure consistency in key variables that affect outcomes in this critically ill population.

## Author contributions

The author confirms being the sole contributor of this work and has approved it for publication.

### Conflict of interest statement

The author declares that the research was conducted in the absence of any commercial or financial relationships that could be construed as a potential conflict of interest.
